# Feeding on Leaves of the Glucosinolate Transporter Mutant *gtr1gtr2* Reduces Fitness of *Myzus persicae*

**DOI:** 10.1007/s10886-015-0641-3

**Published:** 2015-10-28

**Authors:** Svend Roesen Madsen, Grit Kunert, Michael Reichelt, Jonathan Gershenzon, Barbara Ann Halkier

**Affiliations:** DynaMo Center of Excellence, Department of Plant and Environmental Sciences, Faculty of Science, University of Copenhagen, 40 Thorvaldsensvej, DK-1871 Frederiksberg C, Denmark; Max Planck Institute for Chemical Ecology, 07745 Jena, Germany

**Keywords:** Glucosinolates, Aphid performance, Spatial glucosinolate distribution, Transporter mutant, Caged leaves

## Abstract

As aphids are a pest on various crops worldwide, a better understanding of the interaction between aphids and plant host defenses is required. The green peach aphid (*Myzus persicae*) feeds on a variety of plant species, including the model plant *Arabidopsis thaliana* (Arabidopsis), in which glucosinolates function as a major part of the chemical defense. Several studies have shown that glucosinolates play a role in interactions between Arabidopsis and the green peach aphid. In this work, we used a recently identified Arabidopsis glucosinolate transporter mutant (*gtr1gtr2* dKO), with altered glucosinolate content in the vasculature, to investigate the role of defense compound transport in aphid infestation. By monitoring aphid performance on caged leaves and analyzing glucosinolates in leaf tissue and phloem sap, as well as inside aphids, we examined if a change in spatial distribution of glucosinolates within a leaf influences aphid performance. Based on reduced glucosinolate content in the phloem sap of the transporter mutant, we hypothesized that aphids would perform better on *gtr1gtr2* dKO leaves compared to WT. Unexpectedly, aphids performed poorly on *gtr1gtr2* dKO leaves. Our data suggest that higher glucosinolate content in tissues surrounding the phloem of the double transporter mutant may play a role in reducing aphid performance on this genotype.

**Electronic supplementary material:**

The online version of this article (doi:10.1007/s10886-015-0641-3) contains supplementary material, which is available to authorized users.

## Introduction

Aphids constitute a major pest on crops worldwide (Ahuja et al. 2010). The green peach aphid (*Myzus persicae*) feeds on the sugar-rich phloem of a wide array of plant species, including the model plant *Arabidopsis thaliana* (Arabidopsis) (de Vos et al. 2007; Louis 2012). In Arabidopsis, a major part of the chemical defense is constituted by methionine (aliphatic) and tryptophan (indole)-derived glucosinolates (Halkier and Gershenzon 2006; Kliebenstein et al. 2001), which upon plant injury are hydrolyzed rapidly by myrosinases into a multitude of physiologically active products (Halkier and Gershenzon 2006).

Several experiments have shown glucosinolates to affect interactions between Arabidopsis and the green peach aphid. Glucosinolate levels were noted to influence host plant choice, with aphids preferring to feed on Arabidopsis plants with reduced glucosinolate content (Levy et al. 2005). In other studies, aphid reproduction was correlated negatively with the content of aliphatic and indole glucosinolates in Arabidopsis mutants (Mewis et al. 2005, 2006).

Altered glucosinolate levels in response to green peach aphid infestation also have been reported, albeit with conflicting trends: In one study, both aliphatic and indole glucosinolate content increased in Arabidopsis rosettes after one week of aphid feeding (Mewis et al. 2006), whereas a decrease of almost all glucosinolates in Arabidopsis rosettes was observed after three days of aphid exposure in another experiment (Kim and Jander 2007). In the latter study, total glucosinolate content in individual caged leaves infested with aphids was either unchanged or increased, largely due to a local accumulation of 4-methoxyindol-3-ylmethyl glucosinolate (4MI3M) and 8-methylsulfinyloctyl glucosinolate (8-MSO) (Kim and Jander 2007).

While the enzymes involved in glucosinolate biosynthesis have been identified (Jensen et al. 2014; Sonderby et al. 2010), less is known about transport of these compounds. Recently, two glucosinolate-specific importers, GTR1 (NPF2.10) and GTR2 (NPF2.11), were identified in Arabidopsis (Leran et al. 2014; Nour-Eldin et al. 2012). Absence of aliphatic and indole glucosinolates in seeds of the *gtr1gtr2* double knockout (dKO) mutant along with a vascular localization of these transporters demonstrated that GTR1 and GTR2 are essential for long-distance transport of glucosinolates to the seeds, most likely by transporting glucosinolates into phloem companion cells (Nour-Eldin et al. 2012). Investigation of long-distance root-shoot/shoot-root transport of glucosinolates in three-week-old wild-type (WT) and *gtr1gtr2* dKO Arabidopsis plants reported a strong accumulation especially of long-chain, aliphatic glucosinolates (mainly 8-methylsulfinyloctyl (8-MSO)) in rosettes of the glucosinolate transporter mutant (Andersen et al. 2013). Furthermore, 8-MSO and its precursor 8-MTO were found collectively to be 6-fold enriched in the xylem sap of the *gtr1gtr2* dKO mutant compared to WT (Madsen et al. 2014).

Although green peach aphids are phloem feeders, their feeding pattern can be divided into three main phases: a pathway phase, a phloem phase, and a xylem phase (Louis 2012). During the phloem phase, the aphid is feeding mainly from a phloem sieve element. The pathway phase includes intracellular sampling of epidermis and mesophyll cells on the way to the phloem. Xylem phase refers to the time when the insect is ingesting xylem sap, and it is thought to facilitate the uptake of water needed to dilute the high sucrose content of the phloem sap, or to compensate for water loss during periods without feeding. In Arabidopsis, aphids are able to avoid the glucosinolate myrosinase defense since they inflict only minor wounding on the plant while feeding (Barth and Jander 2006), as also evidenced by the fact that intact glucosinolates have been detected in the honey-dew of aphids (Kim and Jander 2007). This suggests that the negative correlation between glucosinolate levels and preferred feeding and fecundity is related to intact glucosinolates, and not their hydrolysis products.

A better understanding of the interaction between aphids and plant host defenses is required, as these insects are worldwide pests on crops. Since glucosinolates are transported via the phloem and xylem saps that aphids feed from, investigation of aphid performance on the *gtr1gtr2* dKO with altered glucosinolate content in the vasculature may improve our understanding of the defensive role of glucosinolates in plant-aphid interactions. In this study, we used the *gtr1gtr2* dKO mutant to investigate whether a change in spatial distribution of glucosinolates within a leaf influences aphid performance. We show that green peach aphid fecundity and survival rate are adversely affected when fed only on the *gtr1gtr2* dKO mutant compared to WT.

## Methods and Material

### Plant Growth Conditions

Seeds of Arabidopsis WT (Col-0) and mutants with T-DNA insertions in both GTR1 (At3g47960) and GTR2 (At5g62680) (*gtr1gtr2* dKO - for more details, see (Nour-Eldin et al. 2012)) were sown on soil in 10 cm pots and cold-stratified at 4 °C for 2 d. Then, the plants were grown in a climate chamber under short day conditions (L:D 10:14 h, 19–21 °C, and 62–70 % relative humidity) for 3.5 wk. until they reached the rosette stage. After this, plants were transferred to long day conditions (L:D 16:8 h) for half a week to adapt to conditions necessary for thriving aphids.

### Leaf Phloem Exudate Collection

Leaves (5 replicates) were cut on the base of the rosette and put into a plastic vial (petiole down) containing 300 μl 20 mM EDTA with 5 mM sodium phosphate buffer (pH 6), and incubated for 1 h in a closed dark box with wet tissues in the bottom to increase humidity (Jander et al. 2004). EDTA was employed to bind Ca^2+^ ions and prevent sieve tube occlusion. However, the contents of cut leaf cells could also leak into the sample. Afterwards, leaves were transferred to new plastic vials containing 300 μl tap water (petiole dipped in water before inserting into vial to rinse off EDTA buffer), and incubated in the same dark box for 4.5 h for phloem sampling. Leaves were weighed for normalization. Water samples including phloem exudates were analyzed for metabolites.

### Glucosinolate Analysis in Phloem and Single Aphids

Aphids (15 replicates) were weighed (around 0.5 mg) and extracted with 0.05 ml of 100 % methanol containing 25 pmol of *para*-hydroxybenzyl glucosinolate (*p*OHB) as an internal standard. The phloem samples were spiked with the same internal standard. Chromatography was performed on an Agilent 1200 HPLC system (Agilent Technologies). Separation was achieved on a Zorbax Eclipse XDB-C18 column (50 × 4.6 mm, 1.8 μm, Agilent). An API 5000 tandem mass spectrometer (Applied Biosystems) equipped with a Turbospray ion source was operated in negative ionization mode. The ion spray voltage was maintained at −4500 eV. The turbo gas temperature was set at 700 °C. Nebulizing gas was set at 60 psi, curtain gas at 25 psi, heating gas at 60 psi, and collision gas at 10 psi. Multiple reaction monitoring (MRM) was used to monitor analyte parent ion → product ion: see (Table 1) for details. Both Q1 and Q3 quadrupoles were maintained at unit resolution. Analyst 1.5 software (Applied Biosystems) was used for data acquisition and processing. Intact glucosinolates were quantified relative to the signal of the internal standard *p*OHB applying the experimentally determined response factors listed in (Table 1).

**Table 1:** Details of analysis of intact glucosinolates (Gls) by LC-MS/MS (HPLC 1200 (Agilent Technologies)-API 5000 (Applied Biosystems)) in negative ionisation mode. Given are the parent-to-product ion transitions used to quantify each compound. LC conditions: flow rate 800 μL/min, formic acid 0.05 % (A), acetonitrile (B): 95 % A (0.5 min), 95–60 % A (3.5 min), 60–0 % A (0.1 min), 0 % A (1.9 min), 0–95 % A (0.1 min), 95 % A (2.4 min). DP, declustering potential; CE, collision energy

Compound	Q1 Mass (Da)	Q3 Mass (Da)	DP	CE	Molar response factor
3-methylsulfinylpropyl-Gls	422.0	95.9	−65	−60	1.04
4-methylsulfinylbutyl-Gls	436.0	95.9	−65	−60	0.79
5-methylsulfinylpentyl-Gls	450.0	95.9	−65	−60	1.24
7-methylsulfinylheptyl-Gls	478.0	95.9	−65	−60	0.93
8-methylsulfinyloctyl-Gls	492.0	95.9	−65	−60	0.32
4-methylthiobutyl-Gls	420.0	95.9	−65	−60	0.74
Indolyl-3-methyl-Gls	447.0	95.9	−65	−60	0.34
4-Methoxy-indolyl-3-methyl-Gls	477.0	95.9	−65	−60	0.25
1-Methoxy-indolyl-3-methyl-Gls	477.0	95.9	−65	−60	0.25
*para*-Hydroxybenzyl Gls	424.0	95.9	−65	−60	1.00

### Sugar Analysis of Phloem Exudates

Sugars in the phloem samples were analyzed directly by LC-MS/MS after a 1:10 (*v*/*v*) dilution in water. Chromatography was performed on an Agilent 1200 HPLC system (Agilent Technologies, Boeblingen, Germany). Separation was achieved on an HILIC-HPLC-column (apHera NH2 Polymer; 15 × 4,6 mm, 5 μm, Supelco). Water and acetonitrile were employed as mobile phases A and B, respectively. The elution profile was: 0–0.5 min, 80 % B in A; 0.5–13 min, 80–55 % B in A; 13–14 min, 55–80 % B in A; and 14–18 min, 80 % B in A. The mobile phase flow rate was 1.0 ml/min. Column temperature was maintained at 25 °C. Liquid chromatography was coupled to an API 3200 tandem mass spectrometer (Applied Biosystems, Darmstadt, Germany) equipped with a Turbospray ion source operated in negative ionization mode. The instrument parameters were optimized by infusion experiments with pure standards (D-(+)-glucose, D-(−)-fructose, sucrose, all Sigma-Aldrich). The ion spray voltage was maintained at −4500 eV. The turbo gas temperature was set at 600 °C. Nebulizing gas was set at 50 psi, curtain gas at 20 psi, heating gas at 60 psi, and collision gas at 5 psi. Multiple reaction monitoring (MRM) was used to monitor analyte parent ion → product ion: *m*/*z* 178.8 → 89.0 (collision energy (CE) -10 V; declustering potential (DP) -25 V) for D-(+)-glucose; *m*/*z* 178.8 → 89.0 (CE -12 V; DP -25 V) for D-(-)-fructose; *m*/*z* 340.9 → 59.0 (CE -46 V; DP -55 V) for sucrose. Both Q1 and Q3 quadrupoles were maintained at unit resolution. Analyst 1.5 software (Applied Biosystems, Darmstadt, Germany) was used for data acquisition and processing. Individual sugars in the sample were quantified by external standard curves generated with a dilution series of authentic standards.

### Amino Acid Analysis of Phloem Exudates

Undiluted phloem samples (245 μl) were mixed with a 13C and 15 N labelled amino acid mix (Algal amino acids 13C,15 N, Isotec, Miamisburg, US) (5 μl) to a concentration of 10 μg/ml. Amino acids in the diluted extracts were analyzed directly by LC-MS/MS. The analysis method was modified from a protocol previously described by Jander et al. (2004). Chromatography was performed on an Agilent 1200 HPLC system (Agilent Technologies, Boeblingen, Germany). Separation was achieved on a Zorbax Eclipse XDB-C18 column (50 × 4.6 mm, 1.8 μm, Agilent Technologies, Germany). Formic acid (0.05 %) in water and acetonitrile were employed as mobile phases A and B, respectively. The elution profile was: 0–1 min, 3 % B in A; 1–2.7 min, 3–100 % B in A; 2.7–3 min 100 % B; and 3.1–6 min 3 % B in A. The mobile phase flow rate was 1.1 ml/min. Column temperature was maintained at 25 °C. Liquid chromatography was coupled to an API 3200 tandem mass spectrometer (Applied Biosystems, Darmstadt, Germany) equipped with a Turbospray ion source operated in positive ionization mode. The instrument parameters were optimized by infusion experiments with pure standards (amino acid standard mix, Fluka, St. Louis, MO, USA). The ionspray voltage was maintained at 5500 eV. Turbo gas temperature was set at 700 °C. Nebulizing gas was set at 70 psi, curtain gas at 35 psi, heating gas at 70 psi, and collision gas at 2 psi. Multiple reaction monitoring (MRM) was used to monitor analyte parent ion → product ion: MRMs were chosen as described previously (Jander et al. 2004), except for Arg (*m*/*z* 175 → 70) and Lys (*m*/*z* 147 → 84). Both Q1 and Q3 quadrupoles were maintained at unit resolution. Analyst 1.5 software (Applied Biosystems) was used for data acquisition and processing. Linearity in ionization efficiencies was verified by analyzing dilution series of standard mixtures (amino acid standard mix, Fluka plus Gln, Asn and Trp, also Fluka). The concentration of the individual labelled amino acids in the mix had been determined by classical HPLC-fluorescence detection analysis after pre-column derivatization with ortho-phthaldialdehyde-mercaptoethanol using external standard curves made from standard mixtures (amino acid standard mix, Fluka plus Gln, Asn and Trp, also Fluka). Individual amino acids in the sample were quantified by the respective 13C, 15 N labeled amino acid internal standard, except for tryptophan and asparagine: Tryptophan was quantified using 13C, 15 N-Phe applying a response factor of 0.42, whereas asparagine was quantified using 13C, 15 N-Asp applying a response factor of 1.0.

### Glucosinolate and Glucosinolate Breakdown Product Analysis of Leaf Extracts

Collected leaves (11 replicates) (~100 mg) were freeze-dried until constant weight and ground to a fine powder. Glucosinolates were extracted with 1 ml of 80 % methanol solution containing 0.05 mM intact *p*-hydroxybenzyl glucosinolate as internal standard. After centrifugation, extracts were loaded onto DEAE Sephadex A 25 columns, and the flow-through of the samples was collected. Bound glucosinolates were treated with arylsulfatase for desulfation (Sigma-Aldrich) overnight. The desulfo glucosinolates were eluted with 0.5 ml water and were separated using high performance liquid chromatography (Agilent 1100 HPLC system, Agilent Technologies) on a reversed phase C-18 column (Nucleodur Sphinx RP, 250 × 4.6 mm, 5 μm, Machrey-Nagel, Düren, Germany) with a water (A)-acetonitrile (B) gradient (0–1 min, 1.5 % B; 1–6 min, 1.5–5 % B; 6–8 min, 5–7 % B; 8–18 min, 7–21 % B; 18–23 min, 21–29 % B; 23–23.1 min, 29–100 % B; 23.1–24 min 100 % B, and 24.1–28 min 1.5 % B; flow 1.0 ml/min). Detection was performed with a photodiode array detector, and peaks were integrated at 229 nm. We used the following response factors: aliphatic glucosinolates 2.0, indole glucosinolates 0.5 (Burow et al. 2006) for quantification of individual glucosinolates.

Flow through fractions (21–23 replicates) from the DEAE Sephadex A 25 columns were collected and diluted in a ratio 1:4 (v:v) with water. Diluted extracts were analyzed by LC-MS using an Agilent 1100 HPLC system (Agilent Technologies, Waldbronn, Germany) coupled to a Bruker Esquire 6000 ion trap mass spectrometer (Bruker Daltonics, Bremen, Germany) operated in alternating ionization mode in the range *m*/*z* 60–1400 (capillary exit voltage, +117; capillary voltage, +4000; nebulizer pressure, 35 psi; drying gas, 11 l/min; gas temperature, 330 °C). Elution was accomplished using a Nucleodur Sphinx RP column (250 × 4.6 mm, 5 μm; Macherey- Nagel, Düren, Germany). Mobile phases were 0.2 % formic acid (v:v) (A) and acetonitrile (B), starting with 100 % A for 5 min, followed by a gradient to 45 % B in 15 min. The subtraction of the mass spectrometer total ion chromatogram of WT plants from that of different mutant plant lines was done using the software package Metabolite Detect 1.1 (Bruker Daltonics, Bremen, Germany) in order to search for metabolites that differ between plant lines. For relative quantification of peak areas of metabolites that differed between plant lines, the respective extracted ion traces were extracted as follows: positive ionization mode: “8-methylsulfinyloctyl amine” *m*/*z* 192; “9-methylsulfinyloctyl nitrile” *m*/*z* 202 (9-methylsulfinylnonyl nitrile =8-methylsulfinyloctyl cyanide).

### Aphid Caging on Leaves

To test for local induction of glucosinolates (and other metabolites) by aphids, 4 WT reared adult aphids were caged on a mature leaf (Fig. S3) for 3 d (11 replicates). Then, caged leaves were collected, aphids removed, and leaves put in liquid nitrogen for further processing (see above). Caged leaves without aphids served as controls.

For the aphid performance assay (15 replicates), 1 adult aphid, reared on a WT plant was caged on a leaf of a WT and a *gtr1gtr2* dKO plant, respectively. One adult aphid reared on a *gtr1gtr2* dKO plant also was caged on a WT and a *gtr1gtr2* dKO leaf. This results in four treatments: WT reared aphids tested on WT ((WT)WT) and on *gtr1gtr2* dKO plants ((WT)dKO), and *gtr1gtr2* dKO reared aphids tested on WT ((dKO)WT) and on *gtr1gtr2* dKO plants ((dKO)dKO). After 3 d, aphid offspring were counted. Aphid infested leaves were collected, aphids were removed, and leaves were put in liquid nitrogen for later metabolite extraction (see above).

### Statistical Analysis

In order to compare glucosinolate concentrations in phloem between WT plants and *gtr1gtr2* dKO plants, the two sample *t*-test with the Welch modification to account for unequal variances was used.

To investigate the influence of aphids and plant genotype on glucosinolate concentration in plant and aphid tissue, we performed a *two-way analysis of variance* (*aov*) if the variances were equal between treatments and residuals were normally distributed. In case of heterogeneity, we used the *generalized least squares* (*gls*) method with the *restricted maximum likelihood estimation* (*REML*) from the nlme package (Pinheiro et al. 2013) to specify the variance structure. The optimal variance structure was chosen based on the Akaike information criterion (AIC). After setting the optimal variance structure, the significance of the explanatory variables (aphid infestation and plant genotype) was evaluated. Therefore, we stepwise removed the explanatory variables from the model, estimated with the *maximum likelihood method* (*ML*), and compared the more complex model with the simplified model using the maximum likelihood ratio test. The minimal model was refitted with the *REML*, and validated for homogeneity of variances and normality of residuals (Zuur et al. 2009). The same procedure was used to look for the influence of rearing and actual test plant on the glucosinolate concentration in aphids.

To test which factor (the rearing plant and the plant where the aphid actually fed) influenced offspring production, we performed a *generalized linear model* with a negative binomial error structure (glm.nb, MASS library (Venables and Ripley 2002)). Significance values were obtained by model simplification and comparison of models using the *maximum likelihood ratio test*.

The concentration of 8-MSO derivatives between WT and *gtr1gtr2* dKO plants was compared with the *Wilcoxon rank sum test*.

All tests were performed in R 3.0.2 (http://www.R-project.org/).

## Results

### Amino Acid, Sugar and Glucosinolate Content in Leaf Phloem Sap

**Fig. 1:**
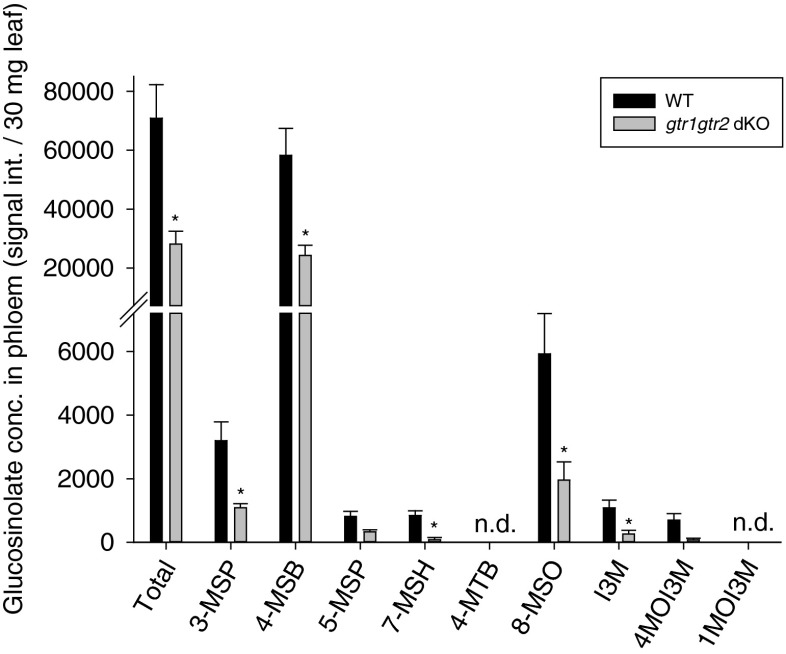
Glucosinolate concentrations in phloem sap from wildtype (WT) and *gtr1gtr2* dKO leaves. Glucosinolates were analyzed in phloem sap exudates. Bars represent means ± SE (*N* = 5). *indicates statistically significant different *gtr1gtr2* dKO glucosinolate concentrations compared to WT (*P* < 0.05), see Methods and Material and (Table S1). Total, total leaf glucosinolates. Glucosinolate abbreviations: 3-MSP, 3-methylsulfinylpropyl; 4-MSB, 4-methylsulfinylbutyl; 4-MTB, 4-methylthiobutyl; 8-MSO, 8-methylsulfinyloctyl; 5-MSP, 5-methylsulfinylpropyl; 7-MSH, 7-methylsulfinylheptyl; I3M, indol-3-ylmethyl; 4MOI3M, 4-methoxyindol-3-ylmethyl; 1MOI3M, 1-methoxyindol-3-ylmethyl; n.d., not detected

Since GTR1 and GTR2 have been suggested to be involved in phloem loading of glucosinolates (Nour-Eldin et al. 2012), we analyzed phloem exudates from WT and *gtr1gtr2* dKO leaves for glucosinolates. We detected reduced levels of all glucosinolates in phloem of the transporter mutant compared to WT plants (Fig. 1). For all but two glucosinolates (5-MSP and 4MOI3M), this reduction was significant (Table S1). 4-Methylsulfinylbutyl (4-MSB) was the dominating glucosinolate in the phloem for both genotypes.

For amino acids, the concentration of alanine in collected *gtr1gtr2* dKO phloem exudates was significantly lower than in WT, whereas a clear trend of reduced amino acid concentration was seen for serine and glutamine in *gtr1gtr2* dKO phloem relative to WT (Fig. S1). Moreover, analysis of phloem sap for fructose, glucose, and sucrose showed that concentrations of sugars were significantly lower in *gtr1gtr2* dKO phloem compared to WT (Fig. S2).

### Glucosinolate Content in Leaf Tissue

**Fig. 2:**
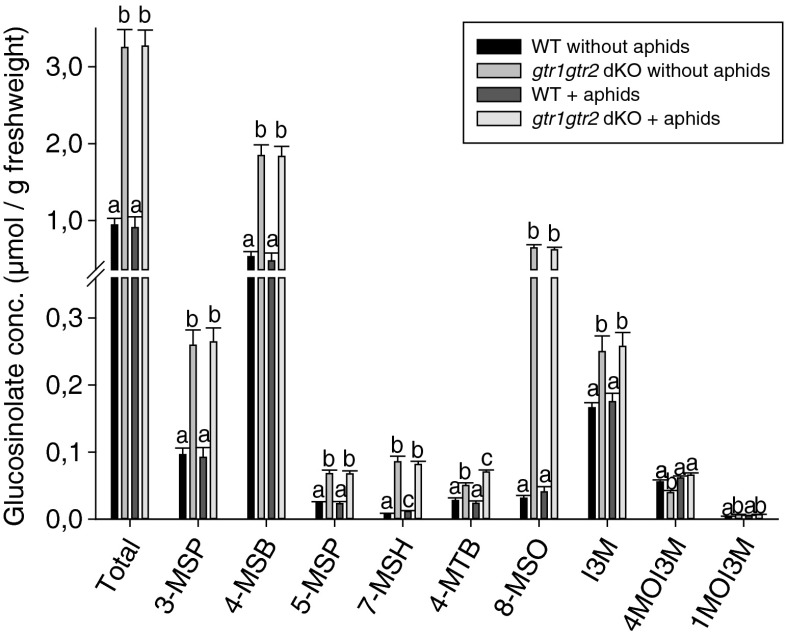
Glucosinolate concentrations in wildtype (WT) and *gtr1gtr2* dKO leaves in the presence and absence of aphids. Leaves caged for 3 d with or without four aphids were analyzed for glucosinolates. Bars represent means ± SE (*N* = 11). Different letters indicate statistically significant different glucosinolate concentrations (*P* < 0.05), see Methods and Material and (Table S2). Total, total leaf glucosinolates. Glucosinolate abbreviations: 3-MSP, 3-methylsulfinylpropyl; 4-MSB, 4-methylsulfinylbutyl; 5-MSP, 5-methylsulfinylpropyl; 7-MSH, 7-methylsulfinylheptyl; 4-MTB, 4-methylthiobutyl; 8-MSO, 8-methylsulfinyloctyl; I3M, indol-3-ylmethyl; 4MOI3M, 4-methoxyindol-3-ylmethyl; 1MOI3M, 1-methoxyindol-3-ylmethyl

We examined whether leaf-caged aphids induced an increase in local leaf accumulation of glucosinolates, and if such an alteration in leaf glucosinolate levels would be dependent on the GTRs. Four aphids were caged on leaves for three days (Fig. S3), after which leaves were analyzed for metabolites. The dominating glucosinolate in leaves was 4-MSB for both genotypes (Fig. 2), as in phloem sap (Fig. 1). As shown in Fig. 2, all glucosinolates were significantly higher in *gtr1gtr2* dKO leaves compared to WT leaves, except for 4MOI3M for which the levels were nearly the same in both genotypes. Aphid infestation led to a small but significant induction of 7-MSH in WT leaves. In *gtr1gtr2* dKO leaves, only, a significant induction due to aphid infestation also was seen for 4-MTB and 4MOI3M (Table S2).

### Feeding on gtr1gtr2 dKO Mutant Leaves Changes Aphid Performance

**Fig. 3:**
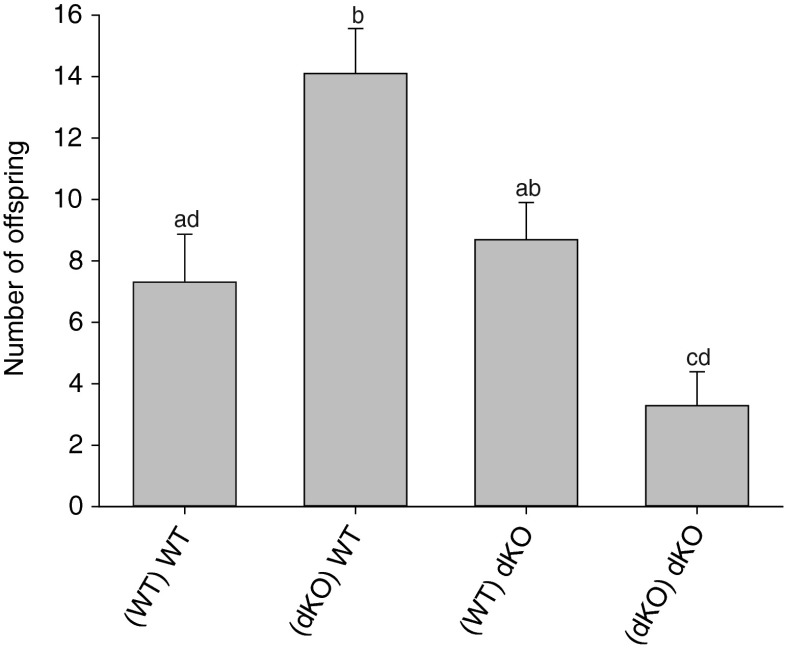
*Myzus persicae* offspring after caging on wildtype (WT) and *gtr1gtr2* dKO leaves. Single aphids, either reared on WT or *gtr1gtr2* dKO plants, were caged on WT and *gtr1gtr2* dKO leaves for 3 d after which their offspring were counted. (WT) WT, aphids were reared on WT plants and produced offspring on WT leaves; (dKO) WT, aphids were reared on *gtr1gtr2* dKO plants and produced offspring on WT leaves; (WT) dKO, aphids were reared on WT plants and produced offspring on *gtr1gtr2* dKO leaves; (dKO) dKO, aphids were reared on *gtr1gtr2* dKO plants and produced offspring on *gtr1gtr2* dKO leaves. Bars represent means **±** SE (*N* = 8–13 adult aphids). Different letters indicate statistically significant different numbers of aphid offspring between the four rearing/caging combinations (*P* < 0.053), see Methods and Material and (Table S3)

Due to the lower levels of glucosinolates in the phloem sap of the transporter mutant, we hypothesized that the green peach aphid would perform better (produce more offspring) on *gtr1gtr2* dKO leaves compared to WT. We tested this by caging single aphids (reared on either WT or *gtr1gtr2* dKO plants) on leaves of both genotypes for three days, after which we counted the aphid offspring. When reared on WT leaves, aphid fecundity was not changed when aphids were then caged on *gtr1gtr2* dKO or WT leaves (Fig. 3). However, when reared on *gtr1gtr2* dKO leaves, the fecundity of aphids then caged on *gtr1gtr2* dKO was strongly reduced (~4.5 fold) compared to fecundity of aphids then caged on WT leaves. In fact, among the four combinations of rearing and leaf caging (see Fig. 3), the highest (WT leaf; ~14) and lowest (*gtr1gtr2* dKO leaf; ~3) number of offspring was counted from leaves with *gtr1gtr2* dKO-reared aphids (Fig. 3).

The proportion of adult aphids that died during the performance experiment (three days) was significantly higher when aphids had been reared on *gtr1gtr2* dKO vs. WT plants, with the highest proportion found when both rearing and caging took place on the transporter mutant (Table 2). Weight of (living) adult aphids did not differ among the various rearing/caging combinations (Fig. S4).

**Table 2:** Proportion of dead adult aphids on caged leaves

(Rearing) and caged leaf	Dead/living (% dead)
(WT) WT	2/15 (13,3)
(*gtr1gtr2* dKO) WT	5/15 (33,3)
(WT) *gtr1gtr2* dKO	2/15 (13,3)
(*gtr1gtr2* dKO) *gtr1gtr2* dKO	7/15 (46,7)
WT reared total	4/30 (13,3)
*gtr1gtr2* dKO reared total	12/30 (40,0)

### Glucosinolate Concentration in Aphids

**Fig. 4:**
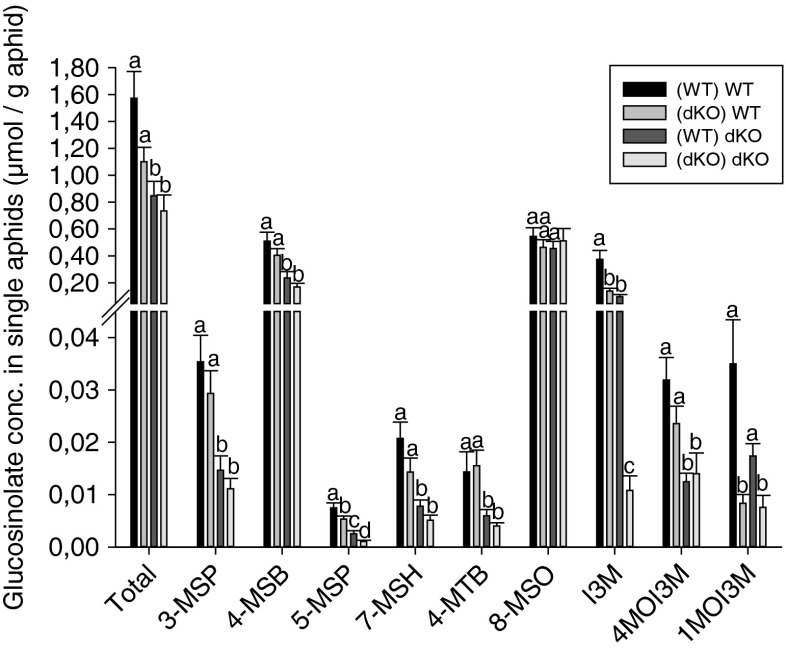
Glucosinolate concentration in adult aphid bodies caged on leaves. Single aphids, either reared on wildtype (WT) or *gtr1gtr2* dKO plants, were caged on WT and *gtr1gtr2* dKO leaves for 3 d after which glucosinolates were analyzed in single aphids. (WT) WT, aphids reared on WT plants and caged on WT leaves; (dKO) WT, aphids reared on *gtr1gtr2* dKO plants and caged on WT leaves; (WT) dKO, aphids reared on WT plants and caged on *gtr1gtr2* dKO leaves; (dKO) dKO, aphids reared on *gtr1gtr2* dKO plants and caged on *gtr1gtr2* dKO leaves. Bars represent means **±** SE (*N* = 8–13 aphids). Different letters indicate statistically significant different glucosinolate concentrations (*P* < 0.05), see Methods and Material and (Table S4). Glucosinolate abbreviations are as described in Fig. 2

To examine whether the reduced glucosinolate content in *gtr1gtr2* dKO leaf phloem (Fig. 1) was reflected in single aphids caged on leaves, we analyzed aphid bodies for glucosinolate content (Fig. 4). The concentration of nearly all glucosinolates in aphids (except for 8-MSO and 1MOI3M) was influenced by which plant the aphid was caged on. Aphids caged on WT leaves generally contained higher glucosinolate concentrations than aphids caged on *gtr1gtr2* dKO plants. The concentration of a few individual glucosinolates in aphids (5-MSP, 7-MSH, I3M, and 1MOI3M) was also influenced by the rearing regime of the aphids: aphids reared on *gtr1gtr2* dKO plants contained significantly less of the above mentioned glucosinolates than aphids reared on WT plants (Table S4). There was a huge reduction in I3M concentration when aphids were reared and caged on *gtr1gtr2* dKO plants compared to when reared on WT plants.

### Accumulation of 8-MSO Glucosinolate Derivatives in gtr1gtr2 dKO Leaves

**Fig. 5:**
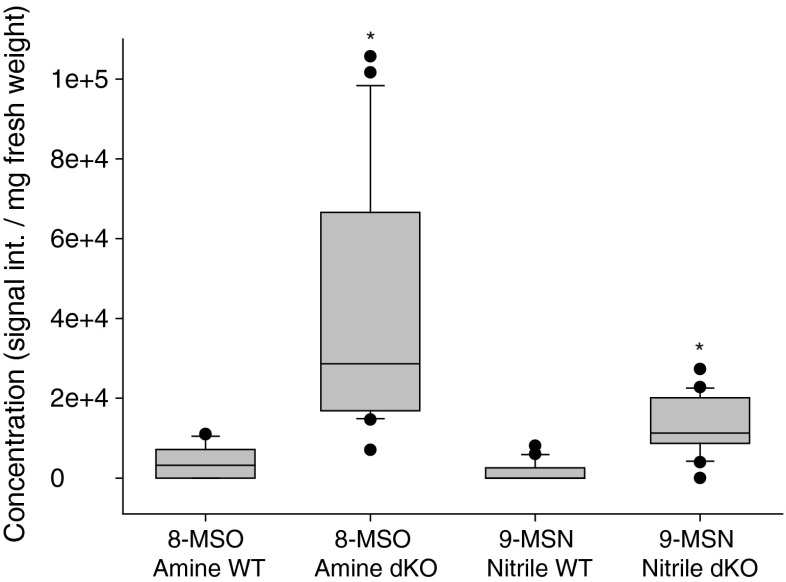
Concentration of 8-MSO derivatives in wildtype (WT) and *gtr1gtr2* dKO leaves. Methanol extracts of leaves (after being caged with one aphid for three days) from WT and *gtr1gtr2* dKO were examined for glucosinolate-derived compounds. 8-methylsulfinyloctyl amine (8-MSO amine) (*m*/*z* = *192*) and 9-methylsulfinylnonyl nitrile (9-MSN nitrile =8-methylsulfinyloctyl cyanide (8-MSO cyanide)) (*m*/*z* = *202*) were identified. Error bars are SE (*N* = 21–23). *indicates statistically significant different *gtr1gtr2* dKO concentrations compared to WT equivalents (*P* < 0.001), see Methods and Material and (Table S5)

To investigate other metabolites that could be involved in aphid performance, WT and *gtr1gtr2* dKO leaf extracts were analyzed for glucosinolate-derived metabolites. Compared to WT, highly increased levels (~10-fold) of the 8-MSO derived metabolites, 8-methylsulfinyloctyl amine (8-MSO amine) and 9-methylsulfinylnonyl nitrile (9-MSN nitrile =8-methylsulfinyloctyl cyanide (8-MSO cyanide)), were identified in *gtr1gtr2* dKO leaves (Fig. 5, Table S5) regardless of aphid infestation (data not shown).

## Discussion

In this work, we used the recently identified Arabidopsis glucosinolate transporter double mutant (*gtr1gtr2* dKO) as a unique tool to investigate the role of intra-leaf glucosinolate distribution and transport in the interaction with the green peach aphid.

Due to their proposed role in phloem loading of glucosinolates (Nour-Eldin et al. 2012), we expected phloem sap of the *gtr1gtr2* dKO to exhibit a decreased glucosinolate content relative to WT phloem, which was indeed the case (Fig. 1). As previous studies have indicated, a defensive role for glucosinolates against aphids (Kim and Jander 2007; Kim et al. 2008; Levy et al. 2005; Mewis et al. 2005, 2006, 2012), we hypothesized that the decrease of glucosinolates in *gtr1gtr2* dKO phloem would cause aphids to perform better on *gtr1gtr2* dKO leaves compared to WT. However, this was not the case (Fig. 3). Aphids tested on *gtr1gtr2* dKO leaves produced similar numbers of offspring (when reared on WT) or even much fewer offspring (when reared on dKO plants), compared with aphids tested on WT plants. Unexpectedly, aphids both reared and caged on *gtr1gtr2* dKO plants produced the least offspring of all rearing/caging combinations. Furthermore, the proportion of adult aphids that died during the performance experiment was higher when these were reared on *gtr1gtr2* dKO plants (40 % for *gtr1gtr2* dKO vs. 13 % for WT) (Table 2). On the other hand, aphids reared on *gtr1gtr2* dKO plants and caged on WT produced the most offspring of all rearing/caging combinations (Fig. 3). In contrast to our hypothesis, one can conclude that both survival and fecundity were adversely affected when aphids had been reared AND caged on the transporter mutant.

An aphid diet is rich in sugars, but relatively poor in amino acids, which are important for aphid growth (de Vos et al. 2007; Louis 2012). Thus, given the important role of amino acids for aphid nutrition, the general reduction of amino acid content in *gtr1gtr2* dKO phloem sap compared to WT (Fig. S1) (rather than the decrease of sugars in *gtr1gtr2* dKO phloem compared to WT (Fig. S2)) may negatively affect the fecundity of aphids reared and caged on *gtr1gtr2* dKO plants. However, aphid proliferation was comparable when feeding on Arabidopsis WT or the *aap6* mutant, which has a significant reduction of amino acid content in phloem sap (Hunt et al. 2010). Thus, lower amino acid availability may not have been responsible for reduced aphid reproduction on *gtr1gtr2* dKO leaves.

Green peach aphid reproduction is not significantly affected by the presence or absence of the TGG1 and TGG2 myrosinases in Arabidopsis (Barth and Jander 2006). This indicates that aphids are able to avoid contact with toxic glucosinolate breakdown products mediated by myrosinases during feeding, and this observation is supported by the presence of intact glucosinolates within aphid bodies (Kim and Jander 2007). Therefore, it can be hypothesized that intact glucosinolates have a defensive role against aphids in Arabidopsis.

As expected, based on phloem sap analysis, glucosinolate concentrations within aphids caged on *gtr1gtr2* dKO leaves were generally lower than in those caged on WT, except for 8-MSO, which had a similar concentration in all aphids (Fig. 4). In most cases, the lowest glucosinolate concentration was detected in aphids reared on *gtr1gtr2* dKO/caged on *gtr1gtr2* dKO, and the highest concentration in aphids reared on WT/caged on WT. As we observed the lowest number of offspring (Fig. 3) and highest mortality rate (Table 2) in aphids both reared and caged on the *gtr1gtr2* dKO, the lower glucosinolate level in these aphids did not fit the hypothesis that intact glucosinolates play an important role in the defense against aphids in Arabidopsis. I3M (and other indole glucosinolates) and breakdown products thereof have been shown to deter aphid feeding more efficiently than aliphatic glucosinolates (Kim and Jander 2007; Kim et al. 2008). As we observed a large reduction of I3M in aphids reared and caged on the *gtr1gtr2* dKO compared to the other rearing/caging combinations (Fig. 4), it was further unexpected that aphids on these plants would perform poorly (Fig. 3). Thus, if glucosinolate levels inside aphids reared and caged on the *gtr1gtr2* could not explain their poor performance, what could be the reason for this observation?

Although the total glucosinolate concentration was significantly lower in *gtr1gtr2* dKO phloem compared to WT (~2.5-fold) (Fig. 1), the opposite was true when comparing glucosinolate concentration in total leaf tissue (~3.5-fold) (Fig. 2), where the concentration of 8-MSO was especially high compared to WT (~18-fold). Thus, in contrast to feeding on *gtr1gtr2* dKO phloem sap that is low in glucosinolate content compared to WT, it can be assumed that aphids living on *gtr1gtr2* dKO plants are encountering higher concentrations of glucosinolates (especially 8-MSO and 4-MSB) during the pathway phase (intracellular sampling of epidermis and mesophyll cells (Louis 2012)) compared to aphids on WT. Furthermore, recent work carried out in our laboratory showed an accumulation of 8-MSO in leaf xylem sap from *gtr1gtr2* dKO relative to WT xylem sap (Andersen et al. 2013; Madsen et al. 2014), indicating that aphids may face high amounts of this glucosinolate during the xylem phase on *gtr1gtr2* dKO plants compared to on WT.

Perhaps the reduced survival rate, fecundity, and glucosinolate content observed for aphids reared and caged on *gtr1gtr2* dKO plants are not a result of the reduced glucosinolate concentration in the phloem of the mutant, but instead reflect that aphids are deterred from feeding as they encounter high concentrations of glucosinolates during the pathway and xylem phases before their stylet reaches the phloem. Alternatively, the reduced amino acid and sugar content in phloem from *gtr1gtr2* dKO leaves compared to WT (Figs. S1, S2) may force aphids to spend more time in the phloem of this genotype. When aphids were reared on *gtr1gtr2* dKO plants and caged on WT leaves, we observed the highest number of offspring (Fig. 3). This may reflect a boost in aphid fitness caused by the lower glucosinolate content in leaf tissue surrounding the phloem, along with a “normal” amino acid and sugar content in the WT phloem sap. When aphids were reared on WT plants and caged on *gtr1gtr2* dKO leaves, we observed a similar number of offspring as aphids reared and caged on WT (Fig. 3). This was unexpected considering the poor performance of aphids caged AND reared on the *gtr1gtr2* dKO. This suggests that aphids reared on the WT can resist the new poor *gtr1gtr2* dKO conditions discussed above. We would, however, expect that if the performance experiment was run for longer than three days, aphid offspring count and mortality rate would eventually decrease and increase, respectively, and reflect the unfavorable aphid environment on the *gtr1gtr2* dKO leaf. These suggest behavioral changes could be investigated by using the Electrical Penetration Graph (EPG) technique (Louis 2012) in future experiments.

The approximately 18-fold increase of 8-MSO in *gtr1gtr2* dKO leaf tissue compared to WT along with the previously reported 8-MSO accumulation in *gtr1gtr2* dKO leaf xylem sap (Andersen et al. 2013; Madsen et al. 2014), suggests a role for this glucosinolate in the poor performance of aphids reared and caged on the transporter mutant. A defensive role of 8-MSO against aphids in Arabidopsis was proposed in previous studies, where 8-MSO accumulated in caged, aphid-infested WT leaves (Ellerbrock et al. 2007; Kim and Jander 2007). However, despite the increased 8-MSO concentration in *gtr1gtr2* dKO leaves compared to WT, aphids contained similar concentrations of this glucosinolate, regardless of rearing and caging (Fig. 4). The potential defensive function of 8-MSO against green peach aphids remains to be determined.

In contrast to phloem and leaf tissue glucosinolate levels (Figs. 1, 2), 4-MSB was not the single dominating glucosinolate inside aphids, as 8-MSO and partly I3M reached similar concentrations as 4-MSB (Fig. 4). Whereas 4-MSB made up 80–90 % of total glucosinolates in the phloem and 50–60 % in leaf tissue, 4-MSB represented only 20–40 % of total glucosinolates inside aphids (Fig. S5). On the other hand, the proportion of 8-MSO inside aphids made up 40–70 % of total glucosinolates, whereas it was only 6–8 % in phloem and 5 (WT)-20 % (*gtr1gtr2* dKO) of total glucosinolates in leaf tissue (Fig. S5). These shifts in glucosinolate proportions from phloem and leaf tissue to aphid may reflect a local accumulation of e.g., 8-MSO at the site of feeding, as has been suggested previously (Kim and Jander 2007); or a selective sequestration (e.g., 8-MSO and 1MOI3M) or discrimination (e.g., 3-MSP, 4-MSB, and 5-MSP) of specific glucosinolates inside the aphid, as has been demonstrated in other crucifer feeding insects, e.g., in the *Phyllotreta striolata* flea beetle (Beran et al. 2014). In contrast to *Phyllotreta striolata*, however, the green peach aphid does not, to our knowledge, harbor its own myrosinase, rendering the reason for sequestration less clear. The difference in glucosinolate proportions inside differently reared/caged aphids most likely reflect altered feeding behaviors of aphids living on either the WT or *gtr1gtr2* dKO, as discussed above.

We investigated whether green peach aphids induced a local glucosinolate increase within infested leaves (Fig. 2), which had been demonstrated in a previous study where long-chain aliphatic and indole glucosinolates (4MOI3M) accumulated in caged Arabidopsis WT leaves exposed to aphids (Kim and Jander 2007). We observed a slight increase of 7-MSH in aphid infested WT plants (Fig. 2). The compounds 4-MTB and 4MOI3M increased only in aphid infested *gtr1gtr2* dKO. Besides the increase of 7-MSH in WT leaves, our data do not correspond to the results reported previously (Kim and Jander 2007). Discrepancies might be explained by differences in experimental design; for example, whereas Kim and Jander (2007) placed 20 aphids on a caged leaf for three days, we placed 4 or 1 aphid(s) in our experiments. Differences in plant growth conditions also may have played a role.

Interestingly, metabolite analysis of leaf extracts revealed a higher (~10 fold) presence of the 8-MSO derivatives, 8-MSO amine, and 9-MSN nitrile (simple nitrile) (= 8-MSO cyanide), in *gtr1gtr2* dKO leaves compared with WT, regardless of aphid infestation (Fig. 5). Simple nitriles are well known glucosinolate hydrolysis products, which are considered less toxic against some insects than the corresponding isothiocyanates (Wittstock and Burow 2010). Their biological function is not fully understood, but a role as signaling compounds toward insects has been proposed. Furthermore, it has been suggested that hydrolysis of nitriles would release the sulfur and nitrogen atoms bound in glucosinolates (Wittstock and Burow 2010). Among other glucosinolate-derived compounds, amines derived from aliphatic glucosinolates were identified in Arabidopsis mutants overexpressing the root myrosinase TGG4, and these compounds were hypothesized to reflect a glucosinolate breakdown pathway in intact plant tissue (Bednarek et al. 2009). Whether the presence of 8-MSO amine and 9-MSN nitrile in leaf samples merely represent the breakdown of glucosinolates during sample preparation, or whether they hold a biological significance is an open question. It is intriguing to speculate that the build-up of 8-MSO amine and 9-MSN nitrile in *gtr1gtr2* dKO leaves represents a way to cope with the accumulation of 8-MSO in this mutant. But this raises the question of why we did not detect 4-MSB derived compounds, as levels of this glucosinolate was higher than 8-MSO in leaves (Fig. 2). Whether the 8-MSO derivatives could affect survival rate and fecundity of aphids on *gtr1gtr2* dKO leaves remains to be investigated.

The *gtr1gtr2* dKO mutant represents a unique opportunity to examine the potential role of glucosinolate transport and local glucosinolate distribution for aphid infestation. However, as mentioned above, further studies including e.g., EPG techniques are required before the role of glucosinolates in the interaction between Arabidopsis and green peach aphids is fully understood. Nevertheless, it appears clear that further investigations of the interplay between this aphid and the *gtr1gtr2* dKO mutant will aid in elucidating the significance of defense compound dynamics involved in Arabidopsis-aphid interactions. Knowledge gained here may be useful in future studies seeking to clarify the implication of defense compound transport in response to insect attack of plants.

## Electronic supplementary material

ESM 1 (DOCX 198 kb)
