# Supplementary material for: Feeding on Leaves of the Glucosinolate Transporter Mutant gtr1gtr2 Reduces Fitness of Myzus persicae
Source: J Chem Ecol. 2015 Oct 28;41(11):975–84. doi: 10.1007/s10886-015-0641-3 (PMC4670620; doi:10.1007/s10886-015-0641-3)
Supplement: Supplementary file 1 — (DOCX 198 kb) [file 10886_2015_641_MOESM1_ESM.docx]

FEEDING ON LEAVES OF THE GLUCOSINOLATE TRANSPORTER MUTANT

*GTR1GTR2* REDUCES FITNESS OF *MYZUS PERSICAE*

*Authors and affiliations*

Svend Roesen Madsen1, Grit Kunert2, Michael Reichelt2, Jonathan Gershenzon2 and Barbara Ann Halkier1*

*1DNRF Center for Dynamic Molecular Interactions (DynaMo), Department of Plant and Environmental Sciences, Faculty of Science, University of Copenhagen, 40 Thorvaldsensvej, DK-1871 Frederiksberg C, Denmark*

*2Max Planck Institute for Chemical Ecology, 07745 Jena, Germany*

***Corresponding author, email: [bah@plen.ku.dk](mailto:bah@plen.ku.dk), phone: +45 35333342

For submission in the **Journal of Chemical Ecology**

SUPPLEMENTARY FIGURES


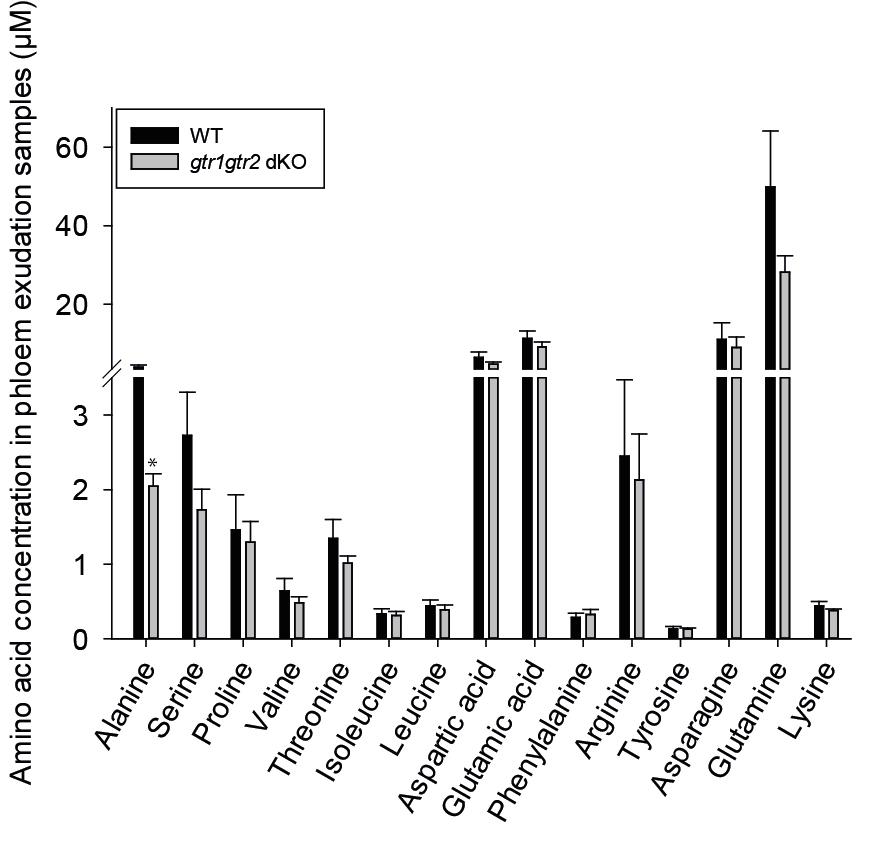


**Amino a cid Method Test value *P*-value**

| alanine | *t*-test | 3,341 | **0,022** |
| --- | --- | --- | --- |
| serine | *t*-test | 1,552 | 0,174 |
| proline | *t* -test | 0,290 | 0,781 |
| valine | *t* -test | 0,855 | 0,429 |
| threonine | *t* -test | 1,285 | 0,256 |
| isoleucine | *t* -test | 0,535 | 0,608 |
| leucine | *t* -test | 0,633 | 0,546 |
| aspartic acid | *t* -test | 1,157 | 0,296 |
| glutamic acid | *t* -test | 0,964 | 0,367 |
| phenylalanine | *t* -test | 0,717 | 0,495 |
| arginine | *t* -test | 0,284 | 0,785 |
| tyrosine | Rank | 14,000 | 0,797 |
| asparagine | *t* -test | 0,405 | 0,698 |
| glutamine | *t* -test | 1,457 | 0,209 |
| lysine | *t* -test | 0,649 | 0,545 |

**Fig. S1** Amino acid concentration in phloem sap exudation samples from WT and *gtr1gtr2* dKO leaves. Phloem sap exudates were analyzed for amino acids. Bars represent mean SE (*N* = 5). *indicates statistically significant different *gtr1gtr2* dKO concentrations compared to WT (*P* < 0.05), all comparisons were done with the two sample *t*-test with Welch modification to account for unequal variances, except for tyrosine, where the Wilcoxon rank sum test was used (see statistical details in the table).

**Sugar  *t* *P*-value** fructose 3,903 **0,010** glucose 3,705 **0,014**

saccharose 3,487 **0,010**


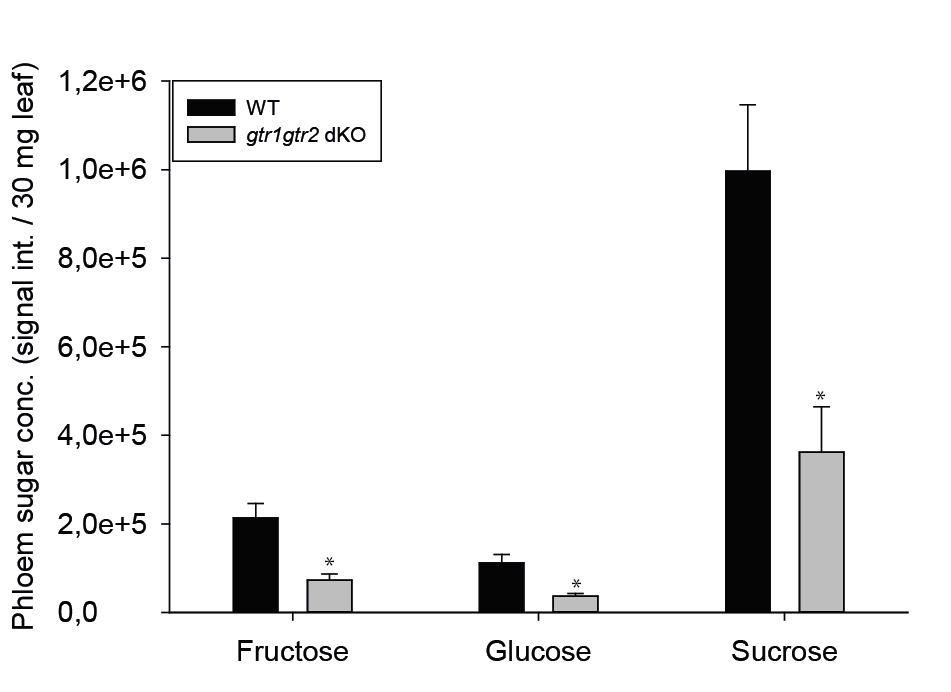


**Fig. S2** Sugar concentration in phloem sap from WT and *gtr1gtr2* dKO leaves. Phloem sap exudates were analyzed for fructose, glucose and sucrose. Bars represent means SE (*N* = 5). *indicates statistically significant different *gtr1gtr2* dKO concentrations compared to WT (*P* < 0.05), two sample *t*-test with Welch modification to account for unequal variances (see statistical details in the table).


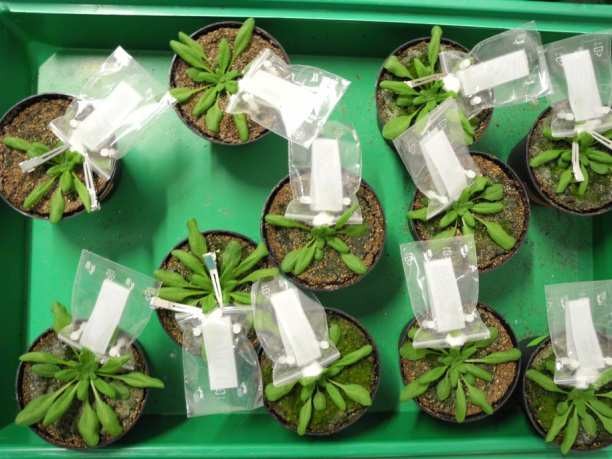


**Fig. S3** Leaf caging setup. Arabidopsis plants with mature leaves caged (in bags) with aphids.


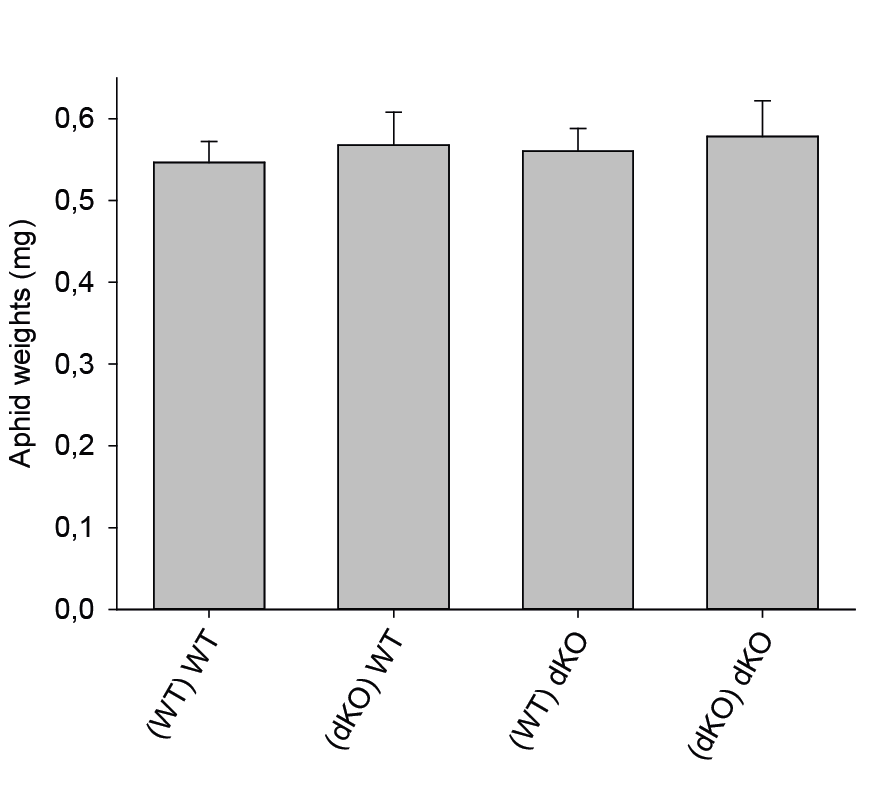


**Test leaf Rearing Interaction**

***F* *P*-value *F* *P*-value *F* *P*-value**

Aphid weight 0,065 0,800 0,609 0,440 0,046 0,831

**Fig. S4** *Myzus persicae* weights after caging on WT and *gtr1gtr2* dKO leaves**.** Single adult aphids, either reared on WT or *gtr1gtr2* dKO plants, were caged on WT and *gtr1gtr2* dKO leaves for 3 d after which adult aphids were weighed. (WT) WT, aphids were reared on WT plants and caged on WT leaves; (dKO) WT, aphids were reared on *gtr1gtr2* dKO plants and caged on WT leaves; (WT) dKO, aphids were reared on WT plants and caged on *gtr1gtr2* dKO leaves; (dKO) dKO, aphids were reared on *gtr1gtr2* dKO plants and caged on *gtr1gtr2* dKO leaves. Bars represent means SE (*N* = 8-13 adult aphids). The aphid weight was neither influenced by the plants the aphids were reared on (*F*=0.609, *P*=0.440) nor by the plants the aphid were caged on (*F*=0.065, *P*=0.800), two-way ANOVA (see statistical details in the table).

.


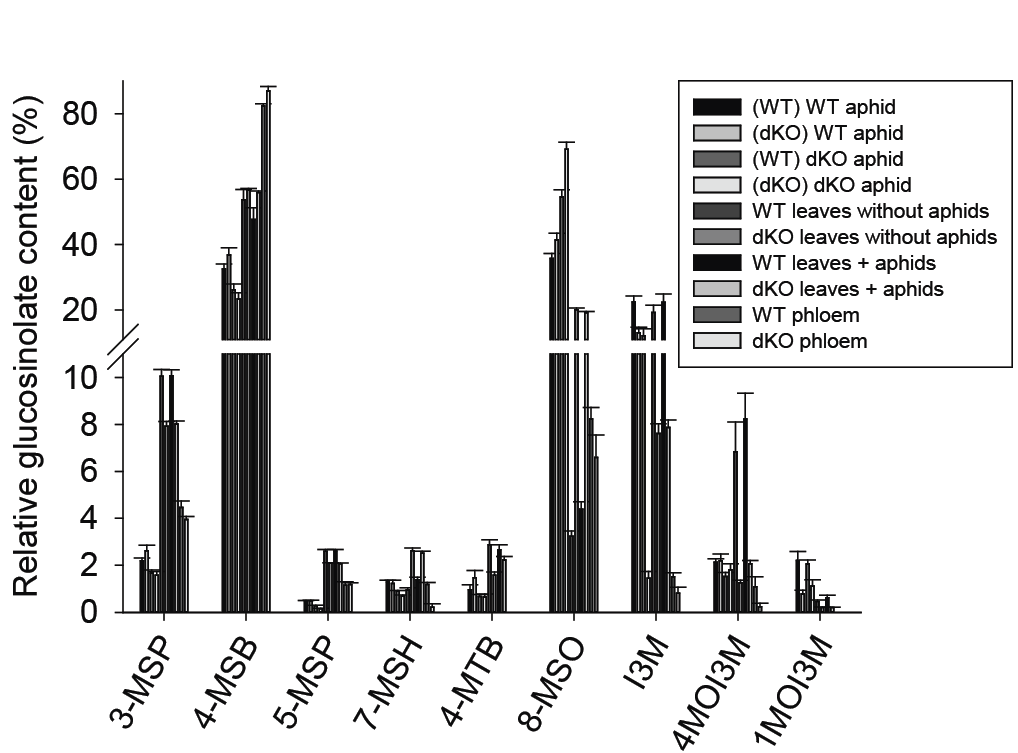


**Fig. S5** Relative glucosinolate content in adult aphid bodies, leaves and phloem sap**.** Single aphids, either reared on WT or *gtr1gtr2* dKO plants, were caged on WT and *gtr1gtr2* dKO leaves for 3 d after which glucosinolates in aphids were analyzed (see Fig. 4). Leaves caged for 3 d with or without four aphids were analyzed for glucosinolates (see Fig. 2). Glucosinolates were analyzed in phloem sap exudates (see Fig. 1). Data are presented as relative glucosinolate content, i.e., as the percentage of a single glucosinolate relative to total glucosinolates in aphid/leaf/phloem. (WT) WT, aphids reared on WT plants and caged on WT leaves; (dKO) WT, aphids reared on *gtr1gtr2* dKO plants and caged on WT leaves; (WT) dKO, aphids reared on WT plants and caged on *gtr1gtr2* dKO leaves; (dKO) dKO, aphids reared on *gtr1gtr2* dKO plants and caged on *gtr1gtr2* dKO leaves. Bars represent means

SE (*N*=8-13 aphids, *N*=11 leaves, *N*=5 phloem samples from five leaves). Glucosinolate abbreviations are as described in Figure 2.

SUPPLEMENTARY TABLES

**Table S1** Statistical analyses of glucosinolate concentration in phloem (Fig. 1). *Welch two sample t-test* (with Welch modification to account for unequal

variances), see Material and Methods.

| **Glucosinolate Te** | **st value** | ***P*-value** |
| --- | --- | --- |
| Total | 3,492 | **0,026** |
| 3-MSP | 3,499 | **0,034** |
| 4-MSB | 3,857 | **0,027** |
| 8-MSO | 2,848 | **0,044** |
| 5-MSP | 2,818 | 0,051 |
| 7-MSH | 4,673 | **0,008** |
| I3M | 3,014 | **0,036** |
| 4MOI3M | 2,963 | 0,052 |

**Table S2** Statistical analyses of glucosinolate concentration in leaves (Fig. 2). *Two-way analysis of*

*variance (aov) and generalized least squares (gls)* methods, see Material and Methods.

**Glucosinolate Method**

**Genotype Aphids Interaction**

|  | | **Test value** | ***P*-value** | **Test value** | ***P*-value** | **Test value** | ***P*-value** |
| --- | --- | --- | --- | --- | --- | --- | --- |
| Total | gls | 54,376 | **< 0.001** | 0,029 | 0,865 | 0,024 | 0,876 |
| 3-MSP | gls | 41,466 | **< 0.001** | 0,017 | 0,895 | 0,068 | 0,795 |
| 4-MSB | gls | 51,845 | **< 0.001** | 0,178 | 0,674 | 0,039 | 0,844 |
| 5-MSP | gls | 49,148 | **< 0.001** | 0,100 | 0,752 | 0,009 | 0,925 |
| 7-MSH | gls | 58,705 | **< 0.001** | 4,199 | **0,041** | 0,579 | 0,447 |
| 4-MTB | aov | 96,117 | **< 0.001** | 5,035 | **0,030** | 12,259 | **0,001** |
| 8-MSO | gls | 71,918 | **< 0.001** | 1,056 | 0,3042 | 0,449 | 0,503 |
| I3M | gls | 18,137 | **< 0.001** | 0,480 | 0,4882 | 0,001 | 0,973 |
| 4MOI3M | aov | 3,189 | 0,082 | 24,650 | **< 0.001** | 10,062 | **0,003** |
| 1MOI3M | aov | 15,08 | **< 0.001** | 0,576 | 0,452 | 0,405 | 0,528 |

**Table S3** Statistical analyses of aphid offspring (Fig. 3).

*Negative binomial model, likelihood ratio test* for optaining P-values. See Material and Methods.

**Test leaf Rearing Interaction**

**LR *P*-value LR *P*-value LR *P*-value**

Aphid number 2,905 0,088 0,035 0,851 12,164 **< 0.001**

**Table S4** Statistical analyses of glucosinolate concentration in aphids (Fig. 4). *Two-way analysis of*

*variance (aov) and generalized least squares (gls) methods*, see Material and Methods.

**Glucosinolate Method**

**Test plant Rearing plant Interaction**

|  | | **Test value** | ***P*-value** | **Test value** | ***P*-value** | **Test value** | ***P*-value** |
| --- | --- | --- | --- | --- | --- | --- | --- |
| Total | gls | 9,017 | **0,003** | 2,999 | 0,083 | 1,795 | 0,180 |
| 3-MSP | gls | 19,041 | **<0.001** | 1,599 | 0,206 | 0,113 | 0,737 |
| 4-MSB | gls | 21,240 | **<0.001** | 3,207 | 0,073 | 0,162 | 0,688 |
| 5-MSP | gls | 33,497 | **<0.001** | 8,035 | **0,005** | 0,330 | 0,566 |
| 7-MSH | gls | 17,300 | **<0.001** | 4,314 | **0,038** | 0,728 | 0,394 |
| 4-MTB | gls | 14,682 | **<0.001** | 1,859 | 0,173 | 0,419 | 0,517 |
| 8-MSO | aov | 0,267 | 0,608 | 0,064 | 0,802 | 1,107 | 0,299 |
| I3M | gls | 30,425 | **<0.001** | 19,930 | **<0.001** | 4,261 | **0,039** |
| 4MOI3M | gls | 16,095 | **<0.001** | 0,396 | 0,529 | 2,167 | 0,141 |
| 1MOI3M | gls | 0,610 | 0,4349 | 13,158 | **<0.001** | 3,309 | 0,069 |

**Table S5** Statistical analyses of 8-MSO derivatives in leaves (Fig. 5). *Wilcoxon rank sum test Welch two sample t-test*, see Material and Methods.

|  | **W** | ***P*-value** |
| --- | --- | --- |
| 8-MSO-Ami ne | 461,0 | **<0.001** |
| 9-MSN Ni tri l e | 449,5 | **<0.001** |
